# Acupuncture recommendations for migraine in headache treatment guidelines: a systematic review

**DOI:** 10.2471/BLT.25.293420

**Published:** 2025-09-29

**Authors:** Shuo Cui, Xiaoyu Wang, Zhenshan Luo, Yaping Liu, Yushan Zhang, Wenqian Ma, Jing Hu, Zhongjie Chen, Jin Huo, Ziwei Song, Qi Gao, Xianghong Jing, Yachan Li, Jingjing Wang

**Affiliations:** a Institute of Acupuncture and Moxibustion, China Academy of Chinese Medical Sciences, 16 Dongzhimennei Nanxiaojie 100700, Dongcheng District, Beijing, China.; b Traditional, Complementary and Integrative Medicine Unit, World Health Organization, Geneva, Switzerland.

## Abstract

**Objective:**

To evaluate the quality of global headache guidelines regarding the recommendation of acupuncture for migraine treatment.

**Methods:**

We searched 31 electronic databases and 15 guideline repositories from inception to October 2024, without language restrictions. We identified 25 guidelines meeting our inclusion criteria, and evaluated these for methodological, reporting and recommendation quality using the Appraisal of Guidelines Research and Evaluation (AGREE) II instrument, Reporting Items for Practice Guidelines in Healthcare (RIGHT) checklist, and AGREE Recommendations Excellence (REX) tool, respectively. We present our findings using descriptive statistics, and assess interrater reliability using intraclass correlation coefficients.

**Findings:**

Of the 25 guidelines analysed, we observed that 40.0% (10/25) recommended acupuncture and 32.0% (8/25) provided conditional recommendations. Of the 18 guidelines recommending acupuncture, 77.8% (14/18) lacked procedural details; only 22.2% (4/18) specified treatment frequency and 16.7% (3/18) described needling techniques. We found that methodological quality was suboptimal, with the domain clarity of presentation scoring highest (75.2%) and applicability lowest (20.3%). We noted that reporting quality was inadequate, particularly in the review and quality assurance domain (18.8%). Only 4.0% (2/25) of guidelines provided high-quality acupuncture recommendations. The 2023 World Federation of Acupuncture–Moxibustion Societies guideline achieved the highest overall quality.

**Conclusion:**

Current global headache guidelines provide limited and inadequately detailed recommendations for acupuncture in migraine treatment, constrained by low methodological quality. Strict adherence to standardized reporting, methodological rigour and recommendation frameworks is essential to generate high-quality evidence, guide clinical decision-making and enhance the global acceptance of acupuncture therapy for migraine.

## Résumé

**Objectif:**

Évaluer la qualité des lignes directrices mondiales sur les céphalées en ce qui concerne la recommandation de l’acupuncture pour le traitement de la migraine.

**Méthodes:**

Nous avons effectué des recherches dans 31 bases de données électroniques et 15 référentiels de lignes directrices depuis leur création jusqu’en octobre 2024, sans restriction linguistique. Nous avons identifié 25 lignes directrices répondant à nos critères d’inclusion et les avons évaluées en termes de qualité méthodologique, de rapportage et de recommandations à l’aide respectivement de l’instrument AGREE II (Appraisal of Guidelines Research and Evaluation), de la liste de contrôle RIGHT (Reporting Items for Practice Guidelines in Healthcare) et de l’outil AGREE REX (Recommendations Excellence). Nous présentons nos résultats à l’aide de statistiques descriptives et évaluons la fiabilité entre les différents évaluateurs à l’aide de coefficients de corrélation intraclasse.

**Résultats:**

Sur les 25 lignes directrices analysées, nous avons observé que 40,0% (10/25) recommandaient l’acupuncture et 32,0% (8/25) fournissaient des recommandations conditionnelles. Sur les 18 lignes directrices recommandant l’acupuncture, 77,8% (14/18) ne fournissaient pas de détails sur les procédures; seules 22,2% (4/18) précisaient la fréquence des traitements et 16,7% (3/18) décrivaient les techniques d’insertion des aiguilles. Nous avons constaté que la qualité méthodologique était sous-optimale, le domaine de la clarté de la présentation obtenant le score le plus élevé (75,2%) et celui de l’applicabilité le plus faible (20,3%). Nous avons remarqué le manque de qualité des rapports, en particulier dans le domaine de l’examen et de l’assurance qualité (18,8%). Seules 4,0% (2/25) des lignes directrices fournissaient des recommandations de haute qualité en matière d’acupuncture. La ligne directrice de la Fédération mondiale des sociétés d’acupuncture-moxibustion de 2023 a obtenu la meilleure note globale en termes de qualité.

**Conclusion:**

Les lignes directrices mondiales actuelles sur les céphalées fournissent des recommandations limitées et insuffisamment détaillées sur l’acupuncture dans le traitement de la migraine, en raison de leur faible qualité méthodologique. Il est essentiel de respecter strictement les normes de rapportage, la rigueur méthodologique et les cadres de recommandation afin de générer des preuves de haute qualité, de guider la prise de décision clinique et d’améliorer l’acceptation mondiale de l’acupuncture dans le traitement de la migraine.

## Resumen

**Objetivo:**

Evaluar la calidad de las guías internacionales sobre cefaleas en lo referente a la recomendación de la acupuntura para el tratamiento de la migraña.

**Métodos:**

Se realizó una búsqueda en 31 bases de datos electrónicas y 15 repositorios de guías desde su creación hasta octubre de 2024, sin restricciones de idioma. Se identificaron 25 guías que cumplían los criterios de inclusión y se evaluaron su calidad metodológica, de presentación y de recomendación mediante los instrumentos Appraisal of Guidelines Research and Evaluation (AGREE) II, Reporting Items for Practice Guidelines in Healthcare (RIGHT) y AGREE Recommendations Excellence (REX), respectivamente. Los resultados se presentan mediante estadística descriptiva y se analizó la fiabilidad interevaluador mediante coeficientes de correlación intraclase.

**Resultados:**

De las 25 guías analizadas, el 40,0% (10/25) recomendaban la acupuntura y el 32,0% (8/25) emitían recomendaciones condicionales. Entre las 18 guías que recomendaban la acupuntura, el 77,8% (14/18) carecían de detalles procedimentales; solo el 22,2% (4/18) especificaban la frecuencia del tratamiento y el 16,7% (3/18) describían las técnicas de punción. La calidad metodológica fue subóptima: la puntuación más alta correspondió al dominio de claridad en la presentación (75,2%) y la más baja al de aplicabilidad (20,3%). La calidad de la presentación resultó insuficiente, especialmente en el dominio de revisión y aseguramiento de la calidad (18,8%). Solo el 4,0% (2/25) de las guías incluían recomendaciones de acupuntura de alta calidad. La guía de 2023 de la Federación Mundial de Sociedades de Acupuntura y Moxibustión alcanzó la puntuación global más alta.

**Conclusión:**

Las guías internacionales sobre cefaleas ofrecen recomendaciones limitadas y poco detalladas sobre la acupuntura en el tratamiento de la migraña, restringidas por una baja calidad metodológica. Es fundamental una adhesión estricta a los estándares de presentación, al rigor metodológico y a los marcos de formulación de recomendaciones para generar evidencia de alta calidad, orientar la toma de decisiones clínicas y fomentar la aceptación mundial de la acupuntura como terapia para la migraña.

## ملخص

الغرض تقييم جودة المبادئ التوجيهية العالمية المتعلقة بالصداع، والتي توصي باستخدام العلاج بالإبر الصينية لعلاج الصداع النصفي.

الطريقة قمنا بالبحث في 31 قاعدة بيانات إلكترونية، و15 مستودعًا للمبادئ للتوجيهية، وذلك منذ إنشائها وحتى أكتوبر/تشرين أول 2024، دون أي قيود لغوية. قمنا بتحديد 25 مبدأً توجيهيًا يتوافق مع معاييرنا للتضمين، وتقييم جودتها من حيث المنهجية والإبلاغ والتوصيات باستخدام أداة تقييم وأبحاث المبادئ التوجيهية (AGREE II)، وقائمة تدقيق عناصر الإبلاغ للمبادئ التوجيهية للممارسة في الرعاية الصحية (RIGHT)، وأداة جودة التوصيات (REX) على التوالي. نحن نقدم نتائج دراستنا باستخدام الإحصاء الوصفي، ونقيّم مدى موثوقية نتائج المحكمين باستخدام معاملات الارتباط داخل المجموعة.

النتائج من بين 25 مبدأً توجيهيًا تم تحليلها، لوحظ أن %40 (10/25) منها أوصت باستخدام العلاج بالإبر الصينية، بينما قدمت %32 (8/25) توصيات مشروطة. من بين 18 مبدأً توجيهيًا توصي باستخدام العلاج بالإبر الصينية، لم تتضمن %77.8 (14/18) تفاصيل حول إجراءات العلاج؛ بينما حددت %22.2 (4/18) فقط عدد جلسات العلاج، ووصف %16.7 (3/18) تقنيات استخدام الإبر. كانت جودة المنهجية غير كافية، حيث حقق وضوح العرض أعلى درجة (%75.2) بينما كانت قابلية التطبيق الأدنى (%20.3). ولاحظنا أن جودة الإبلاغ غير كافية، خاصة في مجال المراجعة وضمان الجودة (%18.8). قدمت %4.0 فقط (2/25) من المبادئ التوجيهية توصيات عالية الجودة للعلاج بالإبر الصينية. حصلت المبادئ التوجيهية للاتحاد العالمي لجمعيات العلاج بالإبر الصينية لعام 2023 على أعلى جودة إجمالية.

الاستنتاج توفر المبادئ التوجيهية العالمية الحالية للصداع توصيات محدودة وغير دقيقة بشأن العلاج بالإبر الصينية للصداع النصفي، نظراً لضعف جودة المنهجية. من الضروري الالتزام الصارم بمعايير الإبلاغ القياسية، والمنهجية الصارمة، وأطر عمل التوصيات، وذلك لإنتاج أدلة عالية الجودة، وتوجيه صنع القرارات الإكلينيكية، وتعزيز قبول العلاج بالإبر الصينية للصداع النصفي عالمياً.

## 摘要

**目的:**

评估关于将针灸作为偏头痛治疗方案建议的全球性头痛治疗指南的质量。

**方法:**

我们搜索了 31 个电子数据库和 15 个指南资源库自创立日起至 2024 年 10 月的数据，没有语言限制。我们确定了 25 个符合纳入标准的指南，并使用《指南研究与评价系统》(AGREE) II 工具、《卫生保健实践指南报告条目》(RIGHT) 清单和《指南建议卓越性评价工具》(AGREE REX)，对这些指南的方法、报告和建议质量进行了评估。我们使用描述性统计数据呈现研究结果，并使用组内相关系数评估了评价者信度。

**结果:**

我们观察到 25 个被分析的指南中，40.0% (10/25) 推荐针灸疗法，32.0% (8/25) 提供有条件的推荐。在 18 个推荐针灸疗法的指南中，77.8% (14/18) 缺乏详细程序信息，仅 22.2% (4/18) 说明了治疗频率，16.7% (3/18) 介绍了针法。我们发现方法质量未达到理想水平，表述清晰度领域得分最高为 (75.2%)，适用性领域得分最低为 (20.3%)。我们注意到报告质量不足，尤其是在评审和质量保证领域 (18.8%)。仅 4.0% (2/25) 的指南提供了高质量的针灸建议。《2023 年世界针灸协会联合会指南》的整体质量最高**。**

**结论:**

当前全球性头痛治疗指南中关于将针灸作为偏头痛治疗方案的建议还比较数量有限，且不够详细，受到方法质量偏低的限制。严格遵守标准化报告、方法严密性和建议框架，对于产生高质量证据、指导临床决策和提高偏头痛针灸疗法的全球接受度而言至关重要。

## Резюме

**Цель:**

Оценить качество международных руководств по лечению головной боли в части рекомендации по применению акупунктуры для терапии мигрени.

**Методы:**

Авторы провели поиск по 31 электронной базе данных и 15 репозиториям руководств от момента создания до октября 2024 года без ограничений по языку материалов. Было выявлено 25 руководств, отвечающих критериям включения, которые затем были проанализированы на предмет качества методологий, отчетности и рекомендаций, при этом использовались такие инструменты оценки, как Appraisal of Guidelines Research and Evaluation (AGREE) II [Анализ качества руководств, научные исследования и оценка], а также контрольный список Reporting Items for Practice Guidelines in Healthcare (RIGHT) [Компоненты для подготовки рекомендаций в здравоохранении] и инструмент AGREE Recommendations Excellence (REX) [Передовой опыт в сфере рекомендаций] соответственно. Результаты представлены с использованием дескриптивной статистики, а надежность оценки, выполненной разными лицами, анализировалась с использованием внутриклассовых коэффициентов корреляции.

**Результаты:**

По наблюдениям авторов, из 25 руководств, 40,0% (10/25) руководств рекомендовали применение акупунктуры и 32,0% (8/25) давали условные рекомендации. Из 18 руководств, рекомендовавших акупунктуру, 77,8% (14/18) содержали недостаточно подробные описания процедуры, только в 22,2% (4/18) указывалась частота лечения, а в 16,7% (3/18) описывалась методика установки игл. Было обнаружено, что методологическое качество рекомендаций не является оптимальным, при этом наивысшие оценки были получены в части четкости изложения (75,2%), а самые низкие – в части применимости (20,3%). Качество отчетности было признано недостаточным, в частности в том, что касалось проверки и обеспечения качества (18,8%). Только 4,0% (2/25) руководств содержали высококачественные рекомендации по применению акупунктуры. Наивысшего качества в целом достигло руководство, выпущенное в 2023 году Всемирной федерацией обществ акупунктуры и моксотерапии.

**Вывод:**

Современные международные руководства по лечению головной боли предоставляют ограниченные и недостаточно подробные рекомендации по применению акупунктуры в лечении мигрени, и их применение затруднено из-за низкого методологического качества. Четкое следование стандартизированным правилам отчетности, методологическая скрупулезность и четкие рамки рекомендаций крайне важны для формирования высококачественной доказательной базы, ее использования в принятии клинических решений и повышения позитивного восприятия акупунктурной терапии мигрени в мировом масштабе.

## Introduction

Migraine is a prevalent and debilitating neurological disorder, affecting approximately 1 billion people globally and representing the second-highest global contributor to disability-adjusted life years in those aged 10–24 years.^1^ According to *The international classification of headache disorders, third edition*,^2^ migraine is divided into six subtypes, of which the most common are migraine without aura and migraine with aura. A typical attack may progress through four phases: the prodrome, aura (if relevant), headache and postdrome. The attack is characterized by a gradually escalating, throbbing pain of moderate to severe intensity, which is often unilateral. This pain typically peaks over several hours and, if untreated, can last 4–72 hours. Associated symptoms include nausea, vomiting, photophobia and phonophobia. As a debilitating disorder, migraine significantly impairs quality of life and imposes substantial economic burdens on health-care systems.^3^ Despite advancements in pharmacological treatments, such as triptans and calcitonin gene-related peptide inhibitors, a considerable proportion of patients experience inadequate relief or intolerable side-effects, highlighting the need for alternative or complementary therapies.^4^

Acupuncture, a key component of traditional Chinese medicine, has gained increasing recognition as a non-pharmacological intervention for migraine management. Clinical trials and systematic reviews have demonstrated its efficacy in reducing migraine frequency, intensity and duration, with minimal adverse effects.^5^^,^^6^ The potential advantages of acupuncture over pharmacological treatments, namely, its minimal side-effects^7^ and long-term efficacy,^8^ make it an attractive alternative to or complementary therapy for migraine management.

However, the integration of acupuncture into routine clinical practice remains inconsistent. A recently conducted survey of clinical practitioners and other relevant stakeholders in China^9^ indicates that only a small proportion of respondents (15/125; 12.0%) have a thorough understanding of clinical practice guidelines for acupuncture; most respondents are aware of the existence of such guidelines, but rarely apply them in actual clinical settings. Factors contributing to this disconnect between knowledge and application include inconsistency in guideline recommendations, and a widely varying quality of evidence supporting its use.^10^

Clinical practice guidelines play a pivotal role in bridging the gap between research evidence and clinical decision-making. High-quality guidelines provide standardized, evidence-based recommendations to optimize patient care and ensure the appropriate use of interventions such as acupuncture.^11^ Nevertheless, the quality of clinical practice guidelines varies widely, influenced by factors such as methodological rigour, transparency and the applicability of recommendations.^12^

Migraine is a condition for which acupuncture has been proven to be particularly effective.^5^^,^^6^^,^^13^ However, there remains substantial international demand for high-quality clinical practice guidelines specifically addressing acupuncture for migraine.^14^ We therefore conducted a systematic review to evaluate the methodological quality, reporting quality and validity of acupuncture-related recommendations in globally available clinical practice guidelines for migraine treatment. We critically assessed the strengths and limitations of existing guidelines, with the aim of providing actionable insights for the future development and implementation of high-quality clinical practice guidelines for the treatment of migraine with acupuncture. 

## Methods

### Search strategy

We registered our systematic review in PROSPERO (CRD420250655920). We searched 31 multilingual electronic databases and 15 specialized websites for articles published from inception to 1 October 2024, without any language restrictions. The 31 databases comprised seven core databases and 25 language-specific databases, and included publications in 13 languages (online repository).^15^ Additionally, we reviewed 15 authoritative websites, including those of the World Health Organization (WHO); World Federation of Acupuncture–Moxibustion Societies; National Institute for Health and Care Excellence; Scottish Intercollegiate Guidelines Network; and other international guideline repositories. Our search strategy incorporated Medical Subject Headings and free-text terms (e.g. “acupuncture,” “migraine,” “headache,” “clinical practice guidelines”). The online repository provides the detailed search methods.^15^

### Screening and inclusion criteria

We included clinical practice guidelines specifically addressing acupuncture for migraine management, and excluded non-guideline publications (for example, research articles related to guideline development or evaluation), guidelines not focused on acupuncture and guidelines unrelated to migraine. In the case of updated guidelines, we only included the latest version. Two authors screened and selected the records independently according to Preferred Reporting Items for Systematic Reviews, using EndNote (Clarivate Analytics, Philadelphia, United States of America). Discrepancies were resolved through consensus or by a third author.

### Data extraction

We used a standardized, pilot-tested data extraction form to collate: (i) basic guideline information, such as, country, publication year, developing organization; (ii) the strength of the evidence; (iii) the strength of the recommendation; and (iv) specific acupuncture recommendations. Two authors independently performed the data extraction using Excel (Microsoft Corporation, Redmond, USA), with cross-verification to ensure accuracy.

### Quality assessment

Five authors independently assessed the methodological quality, reporting quality and quality of acupuncture recommendations of each reviewed guideline using the Appraisal of Guidelines Research and Evaluation II (AGREE II) instrument;^12^ the Reporting Items for Practice Guidelines in Healthcare (RIGHT) checklist;^16^ and the Appraisal of Guidelines Research and Evaluation – Recommendations Excellence (AGREE-REX) tool,^17^ respectively.

The AGREE II instrument^12^ comprises 23 items grouped into six domains: (i) scope and purpose; (ii) stakeholder involvement; (iii) rigour of development; (iv) clarity of presentation; (v) applicability; and (vi) editorial independence. The AGREE-REX instrument^17^ includes nine items categorized within three domains: clinical applicability, values and preferences, and implementability. For both AGREE II and AGREE-REX instruments, we scored each item on a scale from 1 (strongly disagree) to 7 (strongly agree). We calculated the final AGREE II and AGREE-REX domain scores according to the user manuals,^12^^,^^17^ that is, summing the scores for all items within a domain and scaling the total as a percentage of the greatest attainable score for that domain (that is, obtained minus minimum possible score as a percentage of maximum minus minimum possible score).

The RIGHT^16^ checklist includes 35 items organized into seven domains: (i) basic information; (ii) background; (iii) evidence,; (iv) recommendations; (v) review and quality assurance; (vi) funding and declaration and management of interests; and (vii) other information. For each publication, we scored each item as either completely reported (1 point), partly reported (0.5 point) or unreported (0 points).^18^ We calculated individual domain scores as the weighted sum of item scores as a percentage of theoretical maximum domain score.^18^ We report RIGHT checklist results at both the domain level and for individual guidelines.

### Data analysis

We used descriptive statistics to summarize the basic characteristics of the reviewed guidelines. We present continuous variables as mean and standard deviation (SD) and express categorical variables as percentages. 

According to AGREE II,^12^ users can create appropriate thresholds within domain scores for the purposes of quality rating. Following previous publications,^19^^,^^20^ we rated guidelines A if all six domains score 60% or higher, B if four or more domains score 30% or higher, and C if three or more domains score less than 30%. For both AGREE II and AGREE-REX methods of quality assessment, we classified domain quality as good (≥ 80%), acceptable (60–79.9%), poor (40–59.9%) or very poor (< 40%).

We assessed interrater reliability using intraclass correlation coefficients (ICC)^21^^,^^22^ (two-way random model), and interpreted results as very good (> 0.81), good (0.61–0.80), moderate (0.41–0.60), fair (0.21–0.40) and poor (< 0.20). 

### Statistical analysis

We conducted all statistical analyses using Excel, Origin 2024 (OriginLab Corporation, Northampton, USA) and Statistical Package for the Social Sciences version 26.0 (IBM Corporation, Armonk, USA).

## Results

**Fig. 1:**
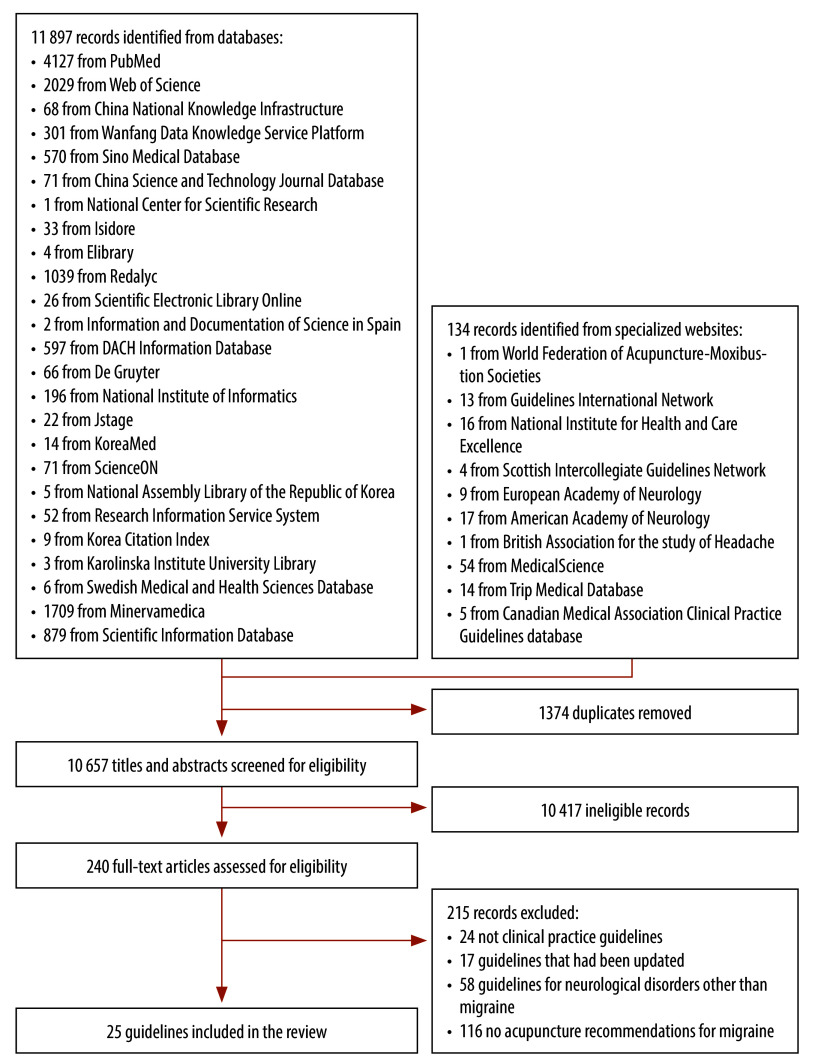
Flowchart depicting the selection of global headache guidelines on acupuncture recommendations for migraine

Our systematic review identified 11 897 records from databases and 134 from specialized websites. After eliminating 1374 duplicates, we screened 10 657 titles and abstracts, retrieving 240 publications for full-text assessment. A total of 25 guidelines met our inclusion criteria (Fig. 1).^23^^–^^47^

### Characteristics of eligible guidelines

Table 1 lists the characteristics of the 25 reviewed clinical practice guidelines.^23^^–^^47^ Our reviewed guidelines were primarily developed by academic groups in China (6/25; 24.0%)^32^^,^^34^^,^^40^^,^^43^^,^^44^^,^^47^ and the USA (6; 24.0%),^25^^,^^27^^,^^28^^,^^35^^,^^39^^,^^45^ followed by the United Kingdom (3; 12.0%)^24^^,^^26^^,^^31^ and Germany (2; 8.0%).^41^^,^^42^ We also reviewed publications based in Belgium,^29^ Canada,^23^ France,^36^ Italy,^30^ Japan,^33^ the Republic of Korea^37^ and the Russian Federation,^38^ and a guideline published by the World Federation of Acupuncture–Moxibustion Societies.^46^ Publications spanned the period 1998–2024, and most guidelines were published in English (14; 56.0%)^23^^–^^28^^,^^30^^,^^31^^,^^35^^,^^36^^,^^39^^,^^41^^,^^42^^,^^45^ or Chinese (6; 24.0%).^32^^,^^34^^,^^40^^,^^43^^,^^44^^,^^47^

**Table 1:** Characteristics of publications included in systematic review of acupuncture recommendations for migraine

Author, year, reference; country (language)	Method of assessment	Whether recommended	Treatment stage	Strength of evidence	Strength of recommendation
Prys-Phillips et al., 1998;^23^ Canada (English)	Canadian Task Force on Preventative Health Care	Recommend	Prophylactic	I (obtained from at least one RCT)	B (fair evidence to support recommendation)
Dowson et al., 2002;^24^ United Kingdom (English)	NR	Conditionally recommend	Prophylactic	NR	NR
Sanders et al., 2005;^25^ USA (English)	NR	Do not recommend	Prophylactic	NR	NR
Scottish Intercollegiate Guidelines Network, 2008;^26^ United Kingdom (English)	Scottish Intercollegiate Guidelines Network	Recommend	Prophylactic	1– (meta-analyses, systematic reviews or RCTs with high risk of bias), 1+ (well conducted meta-analyses, systematic reviews or RCTs with low risk of bias), 1++ (high-quality meta-analyses, systematic reviews or RCTs with very low risk of bias)	B (studies rated 2+, i.e. directly applicable and consistent results)
Gunner et al., 2008;^27^ USA (English)	NR	Do not recommend	Acute and prophylactic	NR	NR
Institute for Clinical Systems Improvement, 2009;^28^ USA (English)	Self-defined	Insufficient evidence to make a definitive recommendation	Prophylactic	A (RCT)	NR
Van Leeuwen et al., 2010;^29^ Belgium (Flemish)	GRADE	Insufficient evidence to make a definitive recommendation	Prophylactic	C (low)	2 (weak)
Sarchielli et al., 2012;^30^ Italy (English)	Self-defined	Conditionally recommend	Acute; prophylactic	NR; A (two or more clinically controlled, randomized, double-blind good clinical practice studies)	IV (drugs of proven efficacy with frequent and/or severe adverse events, or drugs of unproven efficacy or unproven benefit); II (drugs with efficacy of lower statistical significance and with less clinical benefit)
National Institute for Health and Care Excellence, 2012;^31^ United Kingdom (English)	NR	Conditionally recommend	Prophylactic	NR	NR
China Association for Acupuncture and Moxibustion, 2014;^32^ China (Chinese)	GRADE	Recommend	Acute; prophylactic^a^	Filiform needle or electroacupuncture: B (high), C (low), D (very low) and fire needle D; Comprehensive acupuncture: B, C, D	1 (strong), 2 (weak); 1
Hashizume et al., 2016;^33^ Japan (Japanese)	Self-defined	Recommend	Prophylactic	NR	B (recommended)
Huang et al., 2017;^34^ China (Chinese)	Self-defined	Conditionally recommend	Prophylactic	Migraine: B (several supporting but not rigorous RCTs); chronic migraine: C (single trial, no good-quality RCTs)	III (good clinical effect but significant side-effects)
Ha & Gonzalez, 2019;^35^ USA (English)	Strength of recommendations table	Recommend	Prophylactic	A (consistent, good-quality patient-oriented)	NR
Demarquay et al., 2021;^36^ France (English)	Self-defined	Recommend	Acute	NR	Strong (benefits outweigh risks and burdens)
National Institute for Korean Medicine Development; 2021^37^ Republic of Korea (English and Korean)	GRADE	Recommend	Acute; prophylactic	Filiform needle: B (high), C (low); filiform needle and/or electro-acupuncture: B, C	2 (weak)
Ministry of Health of the Russian Federation, 2021;^38^ Russian Federation (Russian)	Self-defined	Conditionally recommend	Prophylactic	1 (high-quality RCTs)	B (recommended and well founded)
Kaiser Permanente Washington, 2018;^39^ USA (English)	NR	Conditionally recommend	Prophylactic	Moderate	NR
Neurologist Branch of Chinese Medical Doctor Association, 2022;^40^ China (Chinese)	GRADE	Recommend	Acute and prophylactic^a^	C (low)	2 (weak)
German Migraine and Headache Society, and German Society of Neurology, 2022;^41^ Germany (English)	NR	Insufficient evidence to make a definitive recommendation	Acute and prophylactic	NR	NR
Diener et al., 2022;^42^ Germany (English)	NR	Insufficient evidence to make a definitive recommendation	Prophylactic	NR	NR
Chinese Association of Integrative Medicine, 2023;^43^ China (Chinese)	GRADE	Recommend	Acute	C (low)	2 (weak)
Chinese Society of Neurology, 2023;^44^ China (Chinese)	Self-defined	Conditionally recommend	Acute and prophylactic	B (at least one high-quality RCT)	II (based on level A or level B strength of evidence)
Sico et al., 2023;^45^ USA (English)	GRADE	Insufficient evidence to make a definitive recommendation	Prophylactic	NR	NR
World Federation of Acupuncture–Moxibustion Societies, 2023;^46^ global (English and Chinese)	GRADE	Conditionally recommend	Acute; prophylactic^a^	Filiform needle: B (high), C (low), D (very low); Filiform needle: B, C, D; electroacupuncture: B, C, D; auricular acupressure: D	2 (weak)
Neurologist Branch of Chinese Medical Doctor Association, 2024;^47^ China (Chinese)	GRADE	Recommend	Acute	B (high)	2 (weak)

GCP: good clinical practice; GRADE: Grading of Recommendations Assessment, Development and Evaluation; NR: not reported; RCT: randomized clinical trial.

^a^ Publications reporting information on acupuncture sessions and courses, such as frequency, duration and timing, and how this may vary according to severity of symptoms.

### Acupuncture recommendations 

Of the analysed guidelines, we observed that 10 (40.0%)^23^^,^^26^^,^^32^^,^^33^^,^^35^^–^^37^^,^^40^^,^^43^^,^^47^ recommended acupuncture; eight (32.0%)^24^^,^^30^^,^^31^^,^^34^^,^^38^^,^^39^^,^^44^^,^^46^ provided conditional recommendations; five (20.0%)^28^^,^^29^^,^^41^^,^^42^^,^^45^ found evidence insufficient; and two (8.0%)^25^^,^^27^ advised against its use. Among those 18 guidelines supporting acupuncture, we noted that 14 (77.8%)^23^^,^^24^^,^^26^^,^^30^^,^^33^^–^^39^^,^^43^^,^^44^ lacked procedural details; only four (22.2%)^31^^,^^32^^,^^40^^,^^46^ specified treatment frequency; and only three (16.7%)^32^^,^^37^^,^^46^ described needling techniques, such as filiform needle, electroacupuncture (full details available in online repository).^15^ Prophylactic treatment was addressed in more than three-quarters of the reviewed guidelines, whereas acute management was addressed in less than one half.

Regarding evidence grading, eight guidelines^29^^,^^32^^,^^37^^,^^40^^,^^43^^,^^45^^–^^47^ used the Grading of Recommendations Assessment, Development and Evaluation (GRADE) tool; three^23^^,^^26^^,^^35^ used other established systems; seven^28^^,^^30^^,^^33^^,^^34^^,^^36^^,^^38^^,^^44^ adopted self-defined systems; and seven^24^^,^^25^^,^^27^^,^^31^^,^^39^^,^^41^^,^^42^ did not specify their grading method (Table 1). 

### Methodological quality 

Our assessment of methodological quality, conducted using the AGREE II instrument, showed good interrater reliability (ICC: 0.776; 95% confidence interval, CI: 0.751–0.799). We noted that mean domain scores varied considerably: clarity of presentation scored the highest (75.2%; acceptable) and applicability the lowest (20.3%; very poor). We rated the quality of three of the remaining four domain scores as poor (Table 2; available online at https://www.who.int/publications/journals/bulletin/). Among the 18 guidelines recommending (or conditionally recommending) acupuncture, we assigned 14^23^^,^^26^^,^^31^^,^^32^^,^^35^^–^^40^^,^^43^^,^^44^^,^^46^^,^^47^ a quality rating of B and four^24^^,^^30^^,^^33^^,^^34^ a quality rating of C; we assigned both guidelines opposing acupuncture a quality rating of C.^25^^,^^27^ We noted that the guideline published by the World Federation of Acupuncture–Moxibustion Societies in 2023^46^ scored highest, and the 2016 Japanese guideline^33^ scored lowest (Table 2).

**Table 2:** Methodological quality of publications included in systematic review of acupuncture recommendations for migraine assessed using the AGREE II instrument

Guideline	Scores for individual domains, %						Overall guideline quality
	Scope and purpose	Stakeholder involvement	Rigour of development	Clarity of presentation	Applicability	Editorial independence	
Prys-Phillips et al., 1998^23^	31.1	32.2	39.6	53.3	0.0	5.0	B
Dowson et al., 2002^24^	54.4	50.0	22.5	46.7	13.3	10.0	C
Sanders et al., 2005^25^	63.3	36.7	26.3	64.4	5.8	1.7	C
Scottish Intercollegiate Guidelines Network, 2008^26^	76.7	56.7	58.8	86.7	5.0	0.0	B
Gunner et al., 2008^27^	72.2	23.3	15.0	57.8	5.0	1.7	C
Institute for Clinical Systems Improvement, 2009^28^	95.6	41.1	23.3	74.4	26.7	66.7	B
Van Leeuwen et al., 2010^29^	87.8	36.7	41.7	81.1	9.2	35.0	B
Sarchielli et al., 2012^30^	46.7	10.0	26.7	67.8	0.8	31.7	C
National Institute for Health and Care Excellence, United Kingdom, 2012^31^	48.9	86.7	89.6	100.0	33.3	55.0	B
China Association for Acupuncture and Moxibustion, 2014^32^	62.2	70.0	46.7	90.0	10.0	0.0	B
Hashizume et al., 2016^33^	44.4	27.8	17.5	52.2	0.8	0.0	C
Huang et al., 2017^34^	58.9	0.0	16.7	96.7	9.2	0.0	C
Ha & Gonzalez, 2019^35^	53.3	15.6	39.6	74.4	33.3	0.0	B
Demarquay et al., 2021^36^	77.8	23.3	43.3	61.1	17.5	83.3	B
National Institute for Korean Medicine Development, 2021^37^	95.6	84.4	85.4	96.7	37.5	71.7	B
Ministry of Health of the Russian Federation, 2021^38^	37.8	56.7	57.5	56.7	15.0	78.3	B
Kaiser Permanente Washington, 2018^39^	67.8	35.6	24.6	86.7	16.7	43.3	B
Neurologist Branch of Chinese Medical Doctor Association, 2022^40^	62.2	33.3	64.6	76.7	32.5	68.3	B
German Migraine and Headache Society, and German Society of Neurology, 2022^41^	45.6	44.4	38.8	76.7	44.2	86.7	B
Diener et al., 2022^42^	91.1	48.9	37.1	61.1	15.0	85.0	B
Chinese Association of Integrative Medicine, 2023^43^	86.7	67.8	63.8	77.8	29.2	91.7	B
Chinese Society of Neurology, 2023^44^	75.6	66.7	43.3	78.9	30.0	63.3	B
Sico et al., 2023^45^	35.6	57.8	50.4	84.4	22.5	51.7	B
World Federation of Acupuncture–Moxibustion Societies, 2023^46^	98.9	90.0	75.4	100.0	58.3	81.7	B
Neurologist Branch of Chinese Medical Doctor Association, 2024^47^	66.7	28.9	62.5	76.7	36.7	81.7	B
**Domain mean (SD)**	**65.5 (20.0)**	**45.0 (23.8)**	**44.4 (21.1)**	**75.2 (15.6)**	**20.3 (15.3)**	**43.7(35.7)**	**NA **

AGREE II: Appraisal of Guidelines for Research and Evaluation; NA: not applicable; SD: standard deviation.

Note: We calculated the scores by summing the scores for all items within a domain and scaling the total as a percentage of the greatest attainable score for that domain (that is, obtained minus minimum possible score as a percentage of maximum minus minimum possible score).^12^ We rated guidelines A if all six domains score 60% or higher, B if four or more domains score 30% or higher, and C if three or more domains score less than 30%.^19^^,^^20^

### Reporting quality 

Our assessment of reporting quality using the RIGHT checklist demonstrated good consistency (ICC: 0.657; 95% CI: 0.630–0.683). Reporting quality across both the domains and the individual guidelines varied considerably: the domain representing basic information scored the highest with 76.7% (standard deviation (SD): 13.4%) and review and quality assurance scored the lowest with 18.8% (SD: 31.5%) (Table 3). We observed that the 2023 World Federation of Acupuncture–Moxibustion Societies guideline^46^ achieved the highest score of 85.9% (SD: 19.7), and a 2016 Japanese guideline^33^ the lowest of 20.0% (SD: 29.8; Table 3).

**Table 3:** Reporting quality of publications included in systematic review of acupuncture recommendations for migraine assessed using the RIGHT checklist

Guidelines	Scores for individual domains, %							Guideline mean, % (SD)
	Basic information	Background	Evidence	Recommendations	Review and quality assurance	Funding and declaration and management of interests	Other information	
Prys-Phillips et al., 1998^23^	66.7	25.0	20.0	50.0	25.0	12.5	0.0	28.5 (22.7)
Dowson et al., 2002^24^	58.3	50.0	10.0	35.7	25.0	0.0	16.7	28.0 (21.2)
Sanders et al., 2005^25^	91.7	43.8	20.0	28.6	0.0	0.0	16.7	28.7 (31.8)
Scottish Intercollegiate Guidelines Network, 2008^26^	66.7	50.0	0.0	14.3	0.0	0.0	16.7	21.1 (26.8)
Gunner et al., 2008^27^	100.0	62.5	40.0	35.7	50.0	12.5	66.7	52.5 (27.7)
Institute for Clinical Systems Improvement, 2009^28^	83.3	81.3	30.0	28.6	0.0	25.0	33.3	40.2 (30.8)
Van Leeuwen et al., 2010^29^	100.0	68.8	40.0	28.6	25.0	12.5	50.0	46.4 (29.9)
Sarchielli et al., 2012^30^	50.0	31.3	20.0	35.7	0.0	12.5	0.0	21.4 (18.8)
National Institute for Health and Care Excellence,United Kingdom, 2012^31^	100.0	100.0	100.0	57.1	75.0	62.5	16.7	73.0 (30.9)
China Association for Acupuncture and Moxibustion, 2014^32^	83.3	100.0	100.0	42.9	0.0	0.0	33.3	51.4 (43.6)
Hashizume et al., 2016^33^	83.3	25.0	10.0	21.4	0.0	0.0	0.0	20.0 (29.8)
Huang et al., 2017^34^	75.0	37.5	20.0	42.9	0.0	0.0	0.0	25.1 (28.5)
Ha & Gonzalez, 2019^35^	66.7	18.8	30.0	42.9	0.0	0.0	16.7	25.0 (23.9)
Demarquay et al., 2021^36^	83.3	87.5	20.0	14.3	0.0	50.0	33.3	48.1 (31.5)
National Institute for Korean Medicine Development, 2021^37^	75.0	50.0	100.0	85.7	100.0	50.0	100.0	80.1 (22.6)
Ministry of Health of the Russian Federation, 2021^38^	66.7	43.8	80.0	71.4	0.0	25.0	33.3	45.7 (28.7)
Kaiser Permanente Washington, 2018^39^	66.7	43.8	20.0	42.9	0.0	0.0	0.0	24.8 (26.8)
Neurologist Branch of Chinese Medical Doctor Association, 2022^40^	66.7	81.3	70.0	92.9	25.0	25.0	50.0	58.7 (26.5)
German Migraine and Headache Society, and German Society of Neurology, 2022^41^	83.3	56.3	20.0	14.3	0.0	50.0	0.0	32.0 (31.8)
Diener et al., 2022^42^	66.7	56.3	20.0	42.9	0.0	100.0	100.0	55.1 (37.8)
Chinese Association of Integrative Medicine, 2023^43^	66.7	68.8	80.0	57.1	0.0	50.0	16.7	48.5 (29.4)
Chinese Society of Neurology, 2023^44^	66.7	62.5	40.0	42.9	0.0	25.0	33.3	38.6 (22.7)
Sico et al., 2023^45^	83.3	12.5	80.0	78.6	25.0	75.0	66.7	60.2 (29.0)
World Federation of Acupuncture–Moxibustion Societies, 2023^46^	91.7	100.0	100.0	92.9	100.0	50.0	66.7	85.9 (19.7)
Neurologist Branch of Chinese Medical Doctor Association, 2024^47^	75.0	87.5	70.0	64.3	0.0	12.5	33.3	48.9 (33.7)
**Domain mean (SD)**	**76.7 (13.4)**	**57.8 (25.9)**	**45.6 (33.6)**	**46.6 (23.4)**	**18.8 (31.5)**	**26.0 (27.7)**	**32.0 (29.6)**	**NA**

NA: not applicable; RIGHT: Reporting Items for Practice Guidelines in Healthcare; SD: standard deviation.

Note: for each publication, we scored each item as either completely reported (1 point), partly reported (0.5 point) or unreported (0 points). We calculated individual domain scores as the weighted sum of item scores as a percentage of theoretical maximum domain score.^18^

### Quality of acupuncture recommendations

Our assessment of the quality of acupuncture recommendations according to the AGREE-REX instrument demonstrated moderate consistency (ICC: 0.570; 95% CI: 0.512–0.628). On average, the quality across all three domains was very poor (Table 4). We observed that only one of the included guidelines^46^ provided high-quality recommendations across all three domains: the 2023 World Federation of Acupuncture–Moxibustion Societies guideline^46^ achieved the highest score of 85.6%. A 2022 German guideline^42^ received the lowest score for quality of recommendations (3.0%) (Table 4). 

**Table 4:** Quality of acupuncture recommendations in publications included in systematic review of acupuncture recommendations for migraine assessed using the AGREE-REX tool

Guideline	Scores for individual domains, %			Overall score
	Clinical applicability	Values and preferences	Implementability	
Prys-Phillips et al., 1998^23^	17.8	6.7	11.7	11.5
Dowson et al., 2002^24^	23.3	2.5	8.3	10.7
Sanders et al., 2005^25^	14.4	0.0	8.3	6.7
Scottish Intercollegiate Guidelines Network, 2008^26^	28.9	5.0	6.7	13.3
Gunner et al., 2008^27^	24.4	4.2	10.0	12.2
Institute for Clinical Systems Improvement, 2009^28^	11.1	0.8	3.3	4.8
Van Leeuwen et al., 2010^29^	8.9	1.7	1.7	4.1
Sarchielli et al., 2012^30^	25.6	2.5	3.3	10.4
National Institute for Health and Care Excellence, 2012^31^	28.9	7.5	5.0	14.1
China Association for Acupuncture and Moxibustion, 2014^32^	33.3	16.7	13.3	21.5
Hashizume et al., 2016^33^	12.2	1.7	5.0	5.9
Huang et al., 2017^34^	25.6	8.3	10.0	14.4
Ha & Gonzalez, 2019^35^	42.2	7.5	16.7	21.1
Demarquay et al., 2021^36^	32.2	20.0	6.7	21.1
National Institute for Korean Medicine Development, 2021^37^	84.4	54.2	70.0	67.8
Ministry of Health of the Russian Federation, 2021^38^	36.7	8.3	11.7	18.5
Kaiser Permanente Washington, 2018^39^	13.3	4.2	5.0	7.4
Neurologist Branch of Chinese Medical Doctor Association, 2022^40^	30.0	6.7	11.7	15.6
German Migraine and Headache Society, and German Society of Neurology, 2022^41^	41.1	26.7	16.7	29.3
Diener et al., 2022^42^	7.8	0.8	0.0	3.0
Chinese Association of Integrative Medicine, 2023^43^	35.6	8.3	5.0	16.7
Chinese Society of Neurology, 2023^44^	44.4	17.5	11.7	25.2
Sico et al., 2023^45^	16.7	0.8	0.0	5.9
World Federation of Acupuncture–Moxibustion Societies, 2023^46^	95.6	77.5	86.7	85.6
Neurologist Branch of Chinese Medical Doctor Association, 2024^47^	27.8	7.5	11.7	15.2
**Domain mean (SD)**	**30.5 (20.8)**	**11.9 (17.8)**	**13.6 (20.2)**	**18.5 (19.0)**

AGREE-REX: Appraisal of Guidelines for Research and Evaluation – Recommendations Excellence; SD: standard deviation.

Note: We calculated the scores by summing the scores for all items within a domain and scaling the total as a percentage of the greatest attainable score for that domain (that is, obtained minus minimum possible score as a percentage of maximum minus minimum possible score).^17^

## Discussion

Guidelines on acupuncture therapy for migraine treatment are notably sparse; only around one tenth of our reviewed publications included details on specific acupuncture techniques and treatment protocols, with the remainder predominantly focused on pharmacological treatments. Few documents report on the frequency and course of acupuncture treatments, reflecting a lack of comprehensive guidance. Acupuncture services generally require advance appointments in most health systems, and the lack of immediate availability remains a key obstacle for acute migraine treatment. Other major barriers include insufficiently qualified practitioners and inconsistent standards and costs, diminishing the real-world clinical utility of acupuncture in acute migraine.

Of our reviewed guidelines, research to measure guideline uptake was only available for a single Chinese publication;^32^ a survey found relatively low utilization rates among both Chinese (55/114; 48.2%) and international experts (13/36; 36.1%).^14^ Although direct studies on the uptake of guidelines are lacking, a survey^48^ indicates limited familiarity among specialists: only 39.7% (23/58) of pain specialists in the United States were aware of the American Headache Society’s acute migraine guideline and 55.7% (34/61) of the American Academy of Neurology’s preventive pharmacotherapy guideline. In China,^9^ 20.8% (26/125) of respondents viewed acupuncture guidelines as impractical and difficult to apply.

We found that the methodological quality of guidelines available for review is suboptimal, consistent with prior AGREE II assessments of migraine guidelines.^49^^,^^50^ We demonstrated that although clearly defined scope and purpose and presentation are common strengths, poor applicability remains a major limitation. Applicability is weakened when guidelines overlook implementation barriers and facilitators, practical tools, resource implications, and monitoring and/or auditing criteria.^19^ In assessing the domain on stakeholder involvement, we found that publications lacked detail on development panel composition and largely omitted patient perspectives. The domain representing rigour of development was compromised by unclear evidence selection criteria, update procedures and evidence grading, reducing overall credibility and applicability. Notably, only a few of the guidelines included methodological or evidence-based medicine experts in their guideline development groups; the absence of such expertise may have contributed to the widespread methodological deficiencies.

We also noted reporting quality as being inadequate. The absence of methodological and evidence-based medical experts in guideline development groups likely contributed to insufficient adherence to reporting standards. The fact that the domain representing review and quality assurance was the least likely to be adequately reported suggests that greater transparency is required in, for example, the development process and handling of feedback. 

Our review highlighted the poor quality of acupuncture recommendations, suggesting inadequate consideration of patient acceptance and policy support (values and preferences) and insufficient specific intervention protocols (implementability). The effectiveness of acupuncture depends on precise technical details such as point selection, stimulation methods, needle retention duration, treatment frequency and course length, but these topics were only covered in a few guidelines. This lack of detail constrains clinical application and effectiveness. 

Given the growing international recognition of acupuncture and the accumulation of high-quality clinical evidence, future migraine or headache treatment guidelines should assign a more clearly defined and prominent role to acupuncture. The development of a unified global standard is both feasible and necessary. With the exception of a traditional Chinese medicine guideline^37^ and a global acupuncture-specific guideline,^46^ all of our reviewed guidelines require substantial improvement across methodological, reporting and acupuncture recommendation domains. Evidently, key factors contributing to the overall low quality and limited applicability of acupuncture guidelines in the treatment of migraine include insufficient methodological rigour; absence of expertise in methods and evidence-based medicine; inadequate review and quality assurance procedures; and inattention to patient values and preferences. Effective dissemination and implementation strategies are also essential to support clinical decision-making and translate evidence into clinically feasible and equitable care.

Our comprehensive approach ensured global coverage of relevant evidence across multiple languages and sources. However, our study also had several limitations. Despite searching databases for minor languages, some region-specific guideline repositories may have been overlooked, limiting the scope of this study to accessible materials. Additionally, although three specialized tools were employed to assess guideline quality, the evaluations remain subjective; other reviewers may have assessed the reviewed publications differently. 

In conclusion, current global headache guidelines provide limited and inadequately detailed recommendations for acupuncture in migraine treatment, constrained by low methodological quality. Strict adherence to standardized reporting, methodological rigour and recommendation frameworks is essential to generate high-quality evidence, guide clinical decision-making and enhance the global acceptance of acupuncture therapy for migraine.
